# Rare association of progressive congenital extrahepatic portosystemic shunt with development of multifocal hepatocellular carcinoma

**DOI:** 10.1259/bjrcr.20150077

**Published:** 2016-07-22

**Authors:** Elliot Landau, Dan Shilo, Blair Rolnick, Jeremy Neuman

**Affiliations:** ^1^Radiology, Staten Island University Hospital, Staten Island, NY, USA; ^2^AUC School of Medicine, Coral Gables, FL, USA

## Abstract

The association between the Abernethy malformation, a rare vascular anomaly in which the portal blood is diverted into the systemic circulation, and development of hepatic tumours is well established. We present a case of multifocal hepatocellular carcinoma (HCC) in the presence of extrahepatic portosystemic shunt with a diminutive portal vein (Type 2 Abernethy malformation). Abdominal ultrasound performed on a 72-year-old female presenting with elevated liver function tests found a 5.6 cm right hepatic lobe mass. Subsequent CT and MRI examinations demonstrated multifocal lesions. A diminutive portal vein was present (transverse diameter of 7 mm) with a large tortuous complex shunt (maximum transverse diameter 2.0 cm) arising at the portal vein bifurcation with branches connecting to the left renal vein and inferior vena cava. Review of a CT examination performed 10 years ago demonstrated a normal-sized portal vein (transverse diameter of 1.5 cm) with a smaller calibre portosystemic shunt (maximum transverse diameter 9 mm). To our knowledge, this is one of the first reports to demonstrate the evolution of progressive portosystemic shunting and the development of HCC.

## Introduction

There is a documented association between congenital extrahepatic portosystemic shunts (CEPS) and the development of both benign and malignant tumours. While the pathogenesis of these lesions remains unclear, nearly one half of all cases of CEPS are associated with nodular liver tumours, most commonly regenerative nodular hyperplasia and focal nodular hyperplasia.^1^ The potential for malignant lesions has been described; however, to this date there have been no clinical or radiographic features that have been associated with malignant processes.^2^ We present a case of an asymptomatic patient with CEPS who developed multifocal hepatocellular carcinoma (HCC) associated with worsening liver function tests and progressive portosystemic shunting. 

## Case report

A 72-year-old female was referred for ultrasound evaluation of the liver secondary to worsening liver function tests. The patient’s history included obesity, for which she had undergone sleeve gastrectomy 2 months ago. Her liver function tests had been slightly elevated in 2012, but had progressed by the time of current presentation (Table 1).

**Table 1.** Comparison of laboratory values from 2012 and 2014. Abnormal values are highlighted in bold

Laboratory tests	September 2012	August 2014	Normal range
Aspartate aminotransferase (U/l)	**46**	**125**	0–41
Alanine aminotransferase (U/l)	24	35	0–45
Alkaline phosphatase (U/l)	**171**	**203**	30–115
Total bilirubin (mg/dl)	**1.3**	**2.5**	0.2–1.2
International normalized ratio	1.1	1.0	0.8–1.2

**Figure 1.**
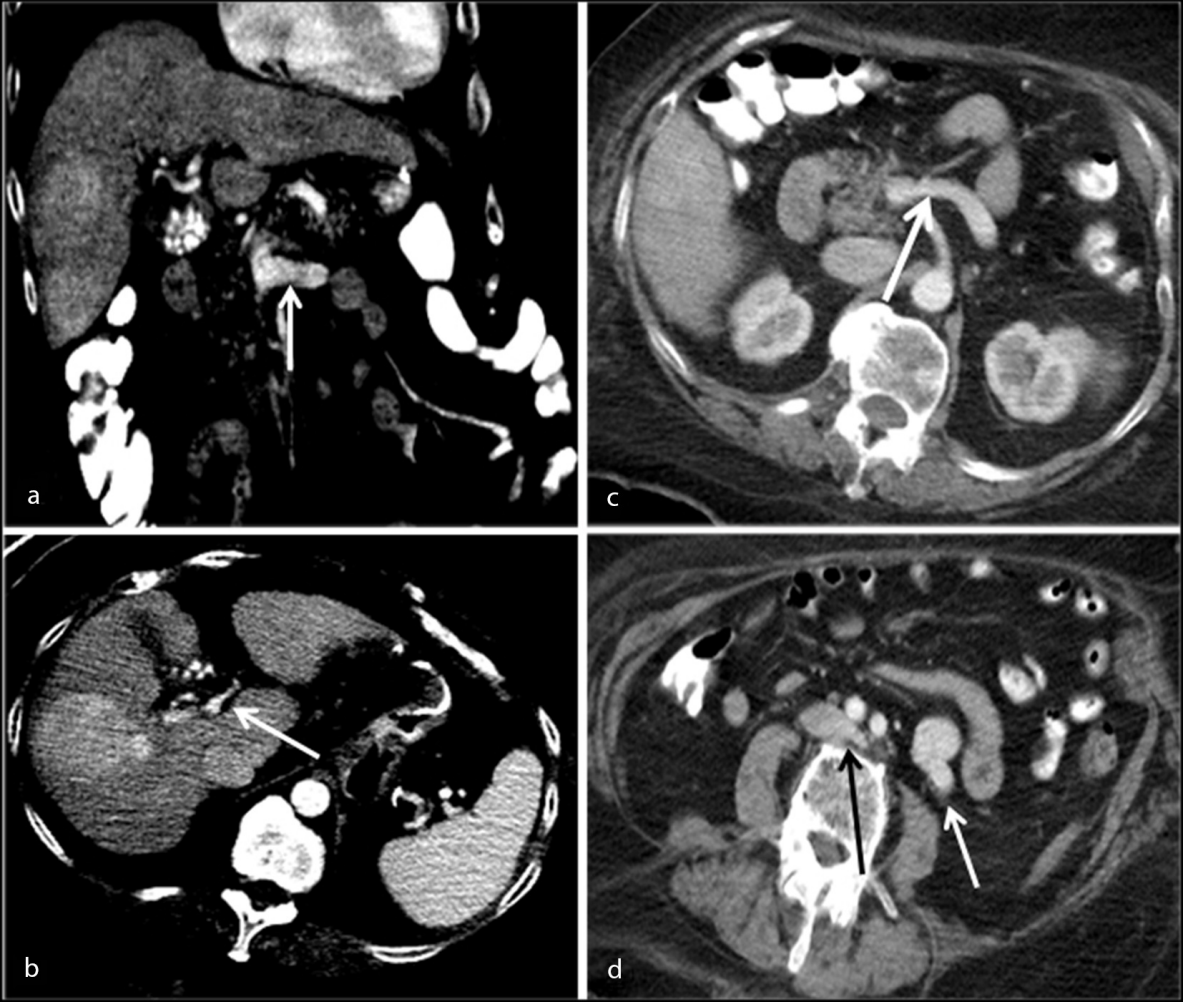
Coronal reformatted contrast-enhanced CT image (a) demonstrates right hepatic lobe lesions. The white arrow focuses on the extra vessel arising from the portal vein. Axial CT image at the level of the main portal vein (b) shows a right hepatic lobe mass with diminutive portal vein (white arrow). Axial image (c) redemonstrates an extra vessel (white arrow) arising from the main portal vein. Axial CT image at a more inferior level (d) shows a dilated tortuous vessel descending along the left para-aortic space (white arrow) with inferior vena cava anastomosis at the level of the aortic bifurcation (black arrow).

**Figure 2.**
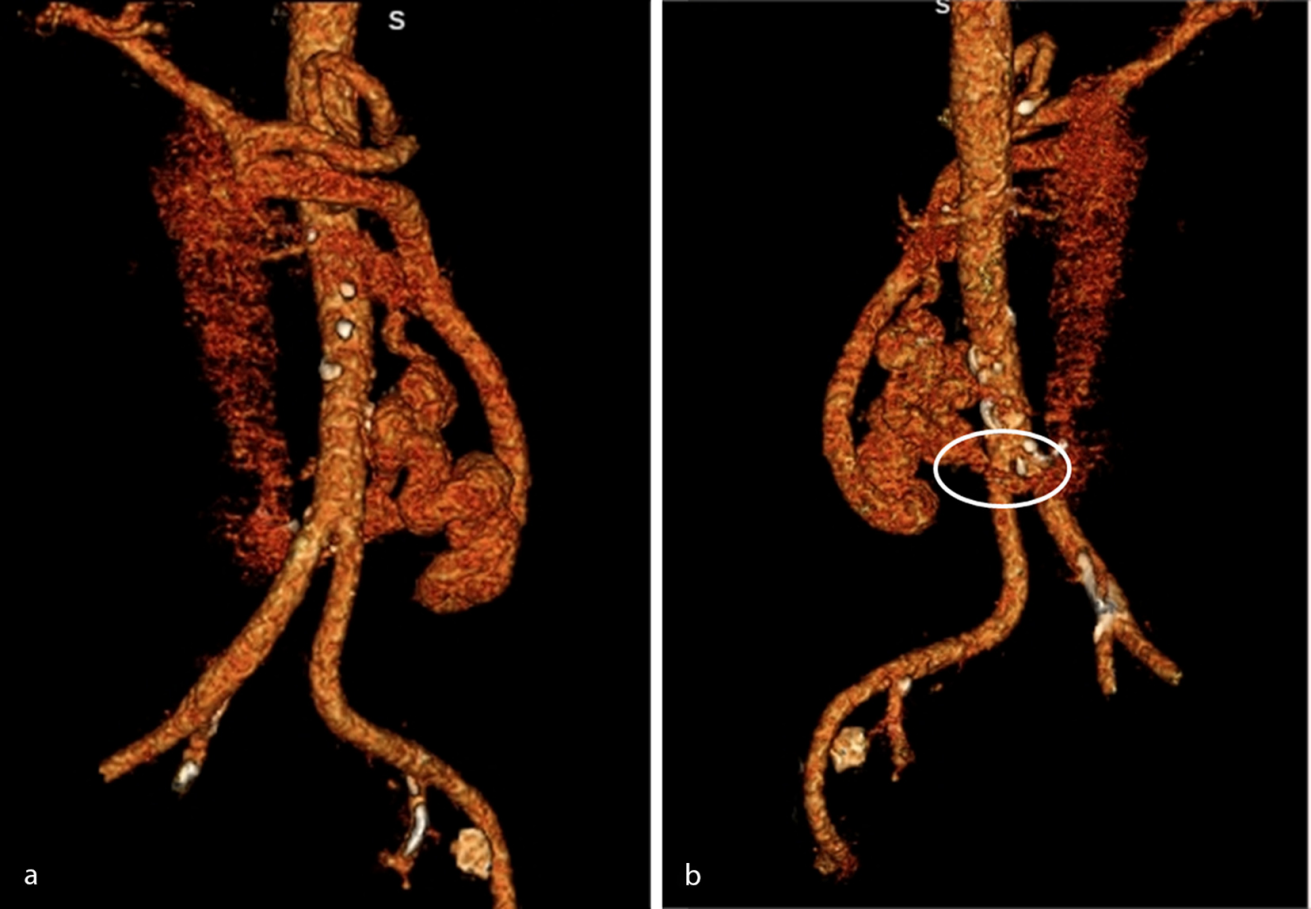
Anterior (a) and posterior (b) three-dimensional reconstruction images demonstrate a dilated vessel arising from the portal vein with a descending tortuous portion along the left para-aortic space. Connection to the inferior vena cava is better appreciated on the posterior projection (white circle).

Ultrasound examination demonstrated a right hepatic lobe hypoechoic solid mass measuring 5.6 cm in transverse diameter. Further evaluation with contrast-enhanced CT scan showed a mildly nodular hepatic surface contour with two heterogeneously enhancing or hyperdense masses containing central areas of diminished attenuation within segments 6 and 7 of the right hepatic lobe (Figure 1). The larger lesion measured 5.3 × 5.7 × 5.1 cm. The portal vein was noted to be diminutive (abnormal for early cirrhosis) with a transverse diameter of 9 mm. A large tortuous complex shunt was noted (maximum transverse diameter of 2.0 cm) to arise from the portal vein bifurcation with connections to the left renal vein and descending along the aorta to communicate with the inferior vena cava at the level of the L2–3 vertebrae (Figures 1 and 2).

**Figure 3.**
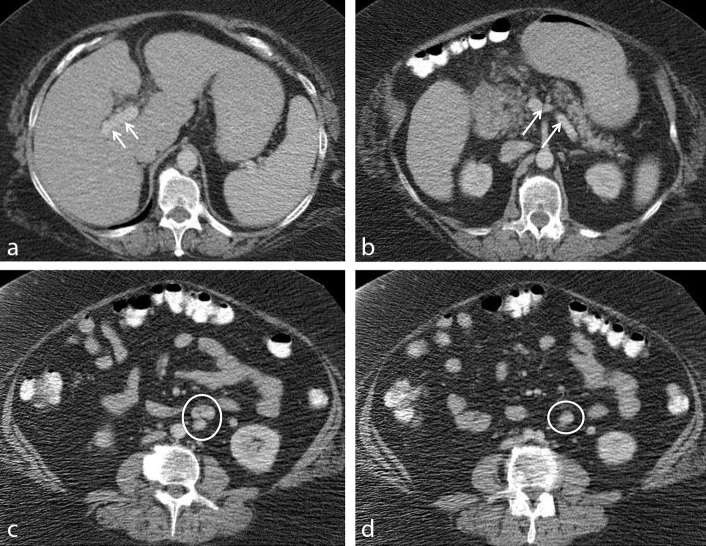
Axial images from contrast-enhanced CT scan performed in September 2004 (a–d). There is a normal calibre portal vein (white arrows, a) as well as an extra vessel arising from the portal vein (white arrows, b). Images (c) and (d) demonstrate tortuous shunt along the left para-aortic space (white circles); the shunt, however, is smaller in calibre compared to its size from 2014.

Review of the patient's chart showed that she had a CT examination performed 10 years ago. Upon review of images from that examination, the liver surface was again noted to be mildly nodular, but without any discrete mass being present. The portal vein was normal in calibre (maximum transverse diameter of 1.5 cm). The described portosystemic shunt was present; however, it was significantly smaller in calibre compared to the current examination, measuring 9 mm in the transverse diameter (Figure 3).

The patient underwent CT-guided biopsy of the larger mass, with pathology results consistent with hepatocellular carcinoma (HCC). The patient is currently undergoing treatment for her disease. The final outcome is yet to be determined.

## Discussion

Congenital extrahepatic portosystemic shunts (CEPS) are vascular malformations that cause either partial or complete diversion of the portal blood flow away from the liver into the systemic veins. First described by John Abernethy in 1793, the disorder has been classified according to the portosystemic anastomosis.^3^ In Type 1 shunts, there is complete agenesis of a true portal vein. The splenic and superior mesenteric veins drain into the systemic circulation either at separate locations (Type 1a) or may form a common trunk with a single systemic anastomosis (Type 1b). These malformations, typically present in the paediatric population, have a very high female predominance (approximately 74%) and are associated with other developmental anomalies, including congenital heart defects, biliary atresia and situs inversus.^4^ In Type 2 shunts, a diminutive portal vein is present with formation of a side-to-side fistula. In contrast to Type 1 anomalies, these patients have no gender predominance and may present at a later stage of life.^5,6^ Several anatomical variations of Type 2 malformations have been described, including portorenal and portocaval connections.^4^

Presenting symptoms of patients presenting later in life include signs of hepatic dysfunction, as in our case, and encephalopathy. Chronic liver dysfunction and cirrhosis are rare but have been reported. The liver disturbance is caused by a lack of hepatocellular nutrition secondary to poor hepatic inflow of blood.^1^ CEPS is usually discovered incidentally during work-up for non- specific liver dysfunction, as in our patient.

One well-established correlation with CEPS is the development of hepatic tumours, which includes both benign and malignant processes. The majority of masses described in patients with CEPS are benign regenerative nodules, followed by focal nodular hyperplasia and benign hyperplastic nodules.^2^ Rare cases of malignant degeneration to HCC have been described. Even more rare cases of hepatoblastoma and hepatic sarcoma have been published as well.^6^ As of yet, the exact pathogenesis of tumour formation has not been confirmed. There are some reports that suggest that tumour development occurs secondary to diversion of splanchnic blood flow from the liver, as substances within the splanchnic venous blood (*i.e.* insulin, glucagon and epidermal growth factor) are essential for hepatocyte function and regeneration.^7^ Furthermore, the increased compensatory hepatic arterial flow could also play a role in neoplastic generation.

There is no consensus as to the proper surveillance of these patients, as well as when to intervene. Some authors suggest routine imaging and biochemical surveillance (alpha-fetoprotein levels), similar to surveillance guidelines for patients with hepatitis B/C infection.^8^ Intervention (including surgical and endovascular procedures) is usually reserved for symptomatic treatment, including patients presenting with encephalopathy, coagulopathy and cardiopulmonary symptoms.

In our patient, sequential CT examinations over 10 years demonstrated progression of the portal venous compromise (decreased portal vein transverse diameter) and increased shunting (increased shunt transverse diameter), with subsequent development of worsening liver dysfunction and multifocal HCC. As with other previously reported cases, there was no evidence of portal hypertension or chronic sequela of cirrhosis. To the best of our knowledge, this is the first report documenting the occurrence of HCC in a case of increased portocaval shunting CEPS coming to light on serial CT imaging.

## Conclusions

Patients found to have CEPS are at a higher risk for developing worsening hepatic dysfunction and tumours. A surveillance program may be beneficial for early detection in these patients. As in our case, progression of portosystemic shunting maybe one early feature associated with deterioration.

## Learning points

1. CEPS are uncommon vascular anomalies classified by the complete absence of the portal vein (Type 1) or partial portal vein perfusion (Type 2).
2. CEPS is associated with hepatic dysfunction, as well as benign and malignant neoplastic development.
3. Progression of portosystemic shunting may be indicative of deterioration and tumour development.

## Consent

Informed consent to publish this case (including images and data) was obtained and is held on record.
